# Identifying the Spatial–Temporal Pattern of Cropland’s Non-Grain Production and Its Effects on Food Security in China

**DOI:** 10.3390/foods11213494

**Published:** 2022-11-03

**Authors:** Jieyong Wang, Chun Dai

**Affiliations:** 1 Institute of Geographic Sciences and Natural Resources Research, Chinese Academy of Sciences (CAS), Beijing 100101, China; 2 Key Laboratory of Regional Sustainable Development Modeling, Chinese Academy of Sciences (CAS), Beijing 100101, China; 3 University of Chinese Academy of Sciences, Beijing 100049, China

**Keywords:** non-grain production of cropland, food security, spatial–temporal pattern, influencing factors, grain production policy

## Abstract

Non-grain production of cropland (NGPCL) is a common phenomenon in the process of rapid urbanization in order to meet the diversified food demand and prosperity of the rural economy. However, excessive NGPCL will threaten grain production. How to control the moderate development of NGPCL in order to achieve the balance between food security and rural development has become a salient issue. In this study, we constructed a framework to measure NGPCL, revealed the spatial–temporal pattern of NGPCL, and then analyzed its influencing factors from the perspective of the human–land relationship. The results indicate that, firstly, the overall degree of NGPCL in China experienced an increase from 0.44 to 0.51, while the gap among cities was consistently enlarging, with the range value increasing from 0.74 to 0.91. Secondly, the spatial pattern of NGPCL was high in the northwest and southeast, and low in the northeast and central regions. The southern economic developed area exhibited the highest increase, while the Inner Mongolia, northwest China, and traditional agricultural areas experienced a decreasing trend in NGPCL. Thirdly, the spatial agglomeration of NGPCL has been intensified, with the Gansu–Xinjiang Desert plateau, southeast coastal economic belt, and urban agglomeration areas exhibiting a “high–high” agglomeration, while the traditional agricultural areas exhibited “low–low” agglomeration. Fourthly, NGPCL is positively correlated with the urbanization rate, land fragmentation, landscape diversity, land price, and grain production policy, while it is negatively linked with the agricultural employment rate, agricultural machinery level, and cultivated land per capita. The findings of this research are not only deepen the understanding of NGPCL, but are also of great significance for policy makers in order to propose targeted control measures.

## 1. Introduction

For many decades, food security has remained a top priority for national security, and constitutes a core component of human development. Despite impressive progress in addressing the basic food needs in the last few decades, important pockets of food insecurity still remain around the world, affecting hundreds of millions of people. Globally, food crop production can only fulfil the demand for the less than one-third of the population [1]. Furthermore, forces originating from population growth and economic development have stimulated food needs, putting increasing pressure on food production [2,3,4,5].

However, global urbanization and industrialization have started to influence the rapid development, which has promoted the drastic transformation of cultivated land use and the off-farm transfer of farmers [6]. A lot of cultivated land has been converted to construction areas, much of which is in areas with a rich soil and high multiple cropping index [7]. In addition to the dominant loss of cultivated land, the recessive loss of the grain production capacity is increasingly difficult to ignore [8]. The emergence of cash crops, new export opportunities for labor-intensive fruits and vegetables, and rising wages have encouraged some of farmers to move out of grain production, resulting in the phenomenon of non-grain production of cropland (NGPCL) [9,10]. Different from non-agriculturalization, NGPCL refers to the phenomenon whereby the cropland intended for grain cultivation is occupied by cash crops, forests, fruit, or the stock-breeding industry, which do not cause a loss of cultivated land, but reduce the food production capacity. As a result, there is mounting pressure to ensure food security [11].

As the most populous country in the world, China has to feed approximately 21% of the world’s population with only 9% of the global cultivated land, which has attracted global attention [12]. Nevertheless, for a long time, China’s per capita grain output has long been hovering right at the standard line of food safety (400 kg/person) set by the FAO, which suggests a grim outlook for national food security. In addition, accelerated urbanization along with explosive economic growth in China has placed increasing pressure on grain production in recent years [13]. The high comparative profit, adjustment of consumption patterns, and acceleration of land transfer have jointly led to a large number of croplands no longer being used for grain production. In East China, a large number of cropland is used to grow tea, citrus, and oil crops; in South China, more and more cropland is used to plant tropical fruits and rubber; in southwest China, planting trees and grass and discarding cultivated land are widely seen, and characteristic crops are generally planted in northwest China. The phenomenon of non-grain of cropland (NGPCL) has proliferated nationwide in China, with the grain acreage being reduced by 970,000 hectares in 2019 alone [14]. Among them, maize plantations lost the most, reaching 874,000 hectares. The planting area of wheat and rice decreased by 538,000 and 495,000 hectares, respectively. Some scholars highlighted that there is not much room for the increase in grain production, and the supply of cropland will become the main restricting factor for agriculture [15]. As a result, NGPCL has become a salient issue in terms of food security and livelihood in China, attracting the attention of governments and researchers.

Given the importance of this issue, a growing body of research has been undertaken on NGPCL, including the influencing factors, impact, and adjustment. There is no clear academic concept of NGPCL and relevant research has focused on crop diversification, multifunctional agriculture, and so on [16,17,18,19]. Drawing on research in these fields, the proportion of the non-grain crop area in the total area is widely used to measure the degree of NGPCL, which ignores the regional differences in the cropping system and underestimates the extent of NGPCL in multi-cropping areas [20,21]. A number of researchers have attempted to disclose the determinants of NGPCL from the perspectives of farmers’ behavior, land economics, and government management [9,10,22]. It is generally believed that plenty of factors, such as rapid farmer situations, topographic conditions, location, and regional policies have an influence on NGPCL [10,23,24,25]. With regards to the impacts of NGPCL, existing studies have proposed that NGPCL renders threats to food production, destroys the soil tillage layer, and causes environmental pollution [21,26]. However, NGPCL could also bring wealth to farmers and meet the diverse food needs of residents [27,28]. Therefore, how to control the moderate development of NGPCL to achieve the balance between food security and rural development has important practical significance. Previous studies have put forward lots of measures to prohibit NGPCL, such as strengthening the control of farmland use, perfecting the land transfer market, increasing grain subsidies, and so on [10,29,30]. As these measures are one-sided and ignore the root cause of NGPCL, they are difficult to be implemented in actual grassroots work. Despite the large number of studies, some limitations in the existing research are worth noting. Firstly, there is a lack of knowledge about the essential characteristics of NGPCL, which is only regarded as a negative phenomenon, ignoring the inevitability and type differences of NGPCL. Secondly, little attention has been paid to the geographical characteristics of NGPCL, thereby failing to reveal the spatial and temporal pattern of NGPCL in China. Thirdly, the analysis of the influencing factors is single and lacks an integrated perspective of the human–land relationship.

Accordingly, this study constructs an index of NGPCL, explores the spatial–temporal dynamics of NGPCL in China at the prefecture level, and quantitatively analyzes its influencing factors from the integrated perspective of the human–land system. These findings will not only deepen our understanding of NGPCL, but also merit particular attention from policymakers aiming to ensure national food security and promote sustainable development in China.

## 2. Theoretical Framework

### 2.1. Connotation Definition

In this study, NGPCL is defined as all farming practices, except grain crop (rice, corn, and wheat) plantations, including both cash crops plantation such as flowers, seedlings, and medicinal herbs, and agricultural production activities using cropland for livestock, poultry, and aquaculture [9,14,27]. NGPCL is essentially a common phenomenon in the process of rapid industrialization and urbanization driven by market demand diversification and economic growth. The quest for a better life has led to higher demands for food diversity and a high income, resulting in diversified and more profitable use of croplands. However, if the NGPCL is overdeveloped, there will be an insufficient grain yield to satisfy the survival of mankind [21]. More seriously, some NGPCL types, such as pond fish farming and nursery plantations, will destroy irrigation facilities and soil tillage layers, making reproduction difficult in the future.

### 2.2. Analysis Framework

**Figure 1:**
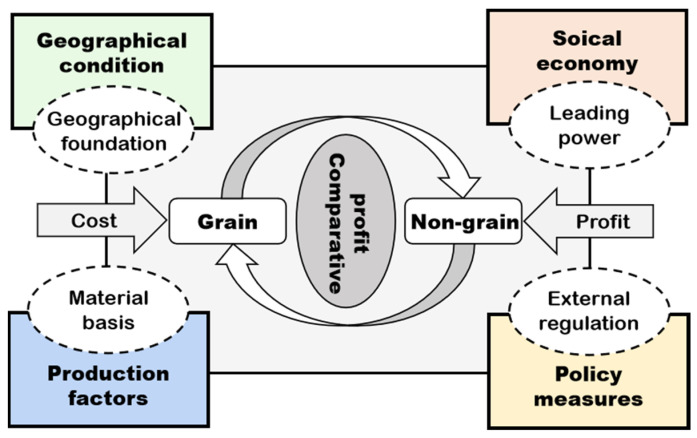
The analysis framework of NGPCL.

NGPCL is a land use transition phenomenon formed by the interaction of natural environments and human activities. Multiple factors, including regional geographical conditions, agricultural production factors, social economy, and grain production policy measures jointly determine the degree and pattern of NGPCL (see Figure 1) [31,32].

The fundamental geographical background predominates the agricultural geographical advantage, thus affecting the area division of labor in agricultural production [33]. Natural geographical factors function as the foundation of cultivated land use and grain production with long-term invariance. The input of agricultural production factors, to a certain extent, can compensate the regional differences brought by the natural environment, providing the material basis for agricultural production and dominating the regional agricultural productivity and agricultural operation mode [34]. Regional economic and social development affects farmers’ production decisions [35]. The development of industrialization and urbanization leads to a change in market demand and enlarges the gap of comparative interests between difference land use, triggering NGPCL [36]. Differentiated grain production policies, which act as the external regulations, change farmers’ motivation for production and thus adjust the spatial pattern of grain production, which is an important external control means of NGPCL [37]. Through a combination of the aforementioned factors, the rising production cost and the continuous low benefit have eliminated the comparative interest in grain production, fundamentally driving the NGPCL.

## 3. Materials and Methods

### 3.1. Methods

#### 3.1.1. Measurement of NGPCL

The index of NGPCL refers to the proportion of cropland used for non-grain purposes in the total cropland of a region, which is calculated as Equation (1):

(1) $$G=1-\frac{L_{g}}{L}$$

where *G* is the index of NGPCL, $L_{g}$ represents the area of cropland occupied by grain crops, and L denotes the total cropland in a region. Furthermore, considering data availability, $L_{g}$ can be decomposed into the ratio of the grain-sowing area to the multiple cropping index. Therefore, the index of NGPCL is the ratio of the grain-sowing area to cropland and the multiple cropping index (Equation (2)).

(2) $$G_{i}=1-\frac{S_{i}}{L_{i}\times M_{i}}$$

where $S_{i}$ denotes the grain-sowing area of the *i*-th unit, $L_{i}$ denotes the cultivated area of the *i*-th unit, and $M_{i}$ denotes the multiple cropping index of the *i*-th unit.

#### 3.1.2. Kernel Density Estimation

The kernel density estimation is a non-parametric estimation method, which is mainly used to estimate the density function of random variables [38]. Its function formula is shown in Equation (3):

(3) $$f\left(x\right)=\frac{1}{nh}\overset{n}{\underset{i=1}{\sum }}k\left(\frac{x-x_{i}}{h}\right)$$

where f(x) is the kernel density estimation function of NGPCL; n is the sample size; and k refers to the kernel function, usually the Gaussian kernel. h is the window width.

#### 3.1.3. Spatial Autocorrelation Analysis

For analysis of the spatial autocorrelation, the global Moran index (*I*) was used to evaluate the global autocorrelation and the Moran local autocorrelation index (LISA), which measures the degree of spatial correlation at each specific site [39]. The calculation of the global Moran index is shown through Equation (4):

(4) $$I=\frac{n\sum_{i=1}^{n}\sum_{j=1}^{n}\left(x_{i}-\bar{x}\right)\left(x_{j}-\bar{x}\right)}{\sum_{i=1}^{n}\sum_{j=1}^{n}w_{ij}\sum_{i=1}^{n}{\left(x_{i}-\bar{x}\right)}^{2}}$$

where n is the number of spatial units; $x_{i}$ and $x_{j}$ refer to value of the attribute *X* considered in areas *i* and *j*, respectively; $\bar{x}$ denotes the average value of the attribute *X* in the study region; and $w_{ij}$ denotes the *i*-th row and *j*-th column elements of the spatial weight matrix. In this study, the criterion of Rook contiguity was used. The Rook matrix defines neighbors as having common edges, but no common vertices. Rook contiguity is a spatial matrix assignment in which dummy variables of two regions with common boundaries are set to 1 and 0 otherwise [40]. The formula of LISA is Equation (5):

(5) $$I_{i}=\frac{x_{i}-\mu }{\sigma_{n}^{2}}\sum_{j=1}^{n}w_{ij}\left(x_{j}-\mu \right)$$

where $\sigma_{n}^{2}$ refers to the variance of the variable *X* under study in the *n* units, and μ denotes the average of *n* units. LISA serves to identify the patterns of local spatial fragmentation, extreme spatial value, and to capture the patterns of local association.

#### 3.1.4. Spatial Econometric Model

Spatial econometrics apply the basic idea of geography to solve the spatial problems of regional economic data. This paper empirically tests the influencing factors of NGPCL through the ordinary least square regression model (OLS), spatial lag model (SLM), and spatial error model (SEM). The empirical models are constructed in Equation (6):

(6) Y=βX+ε

where Y denotes NGPCL, X denotes explanatory variables, *β* is the regression coefficient of explanatory variable, and ε is the error term.

Equation (6) is the ordinary least square regression model. Considering the spatial effects of NGPCL, we established a spatial lag model (SLM) and spatial error model (SEM). SLM, also called the spatial autoregressive model (SAR), is mainly used to analyze the spillover effects among different variables in a certain region. SEM refers to the spatial econometric model in which the error term is introduced into the spatial autocorrelation. Equations (7) and (8) are for SLM and SEM, respectively.

(7) Y=α+ρWY+βX+ε

(8) Y=α+βX+ϕ

(9) ϕ=λWϕ+ε

where α denotes the constant term; ρ and λ refer to the regression coefficients of SLM and SEM, respectively; W denotes the spatial weight matrix; and ϕ is the spatial autocorrelation error term.

### 3.2. Variable Selection

**Figure 2:**
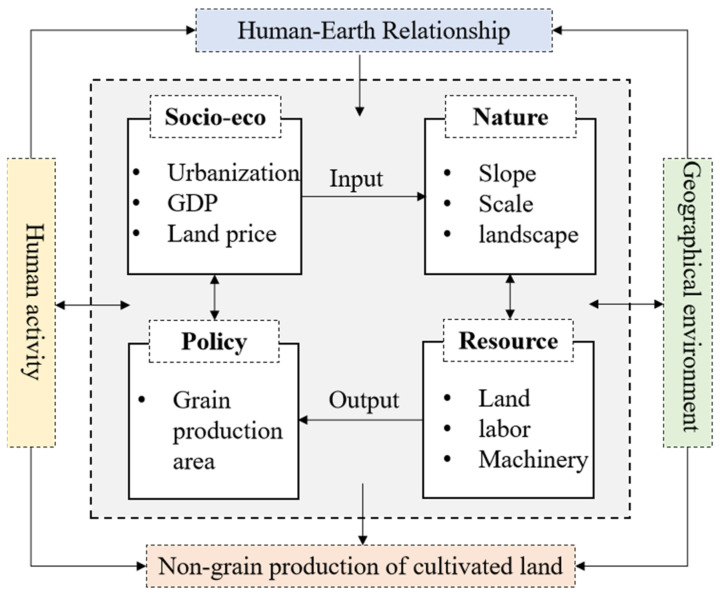
Framework of the influencing factors of NGPCL.

This study refers to previous research results, based on the research framework (see Figure 1) and combined with the availability of data, to select the primary driving factors for NGPCL. These factors can better explain the impetus driving NGPCL from different viewpoints, including natural geographical environments, agricultural production resources, socio-economic development, and grain production policies (see Table 1 and Figure 2).

*Natural geographical environment:* Natural geographical environments generally dominate the direction of cropland utilization [41]. The geographical conditions, including the topography, land morphology, and landscape pattern, jointly influence the scale of agricultural cultivation and the distribution of crop species [42]. In this study, slope, cultivated land fragmentation, and landscape diversity are selected as the natural geographical factors.

*Agricultural production factors:* Agricultural production factors, including land, labor, and agricultural technology, are the material basis of land use [15,43]. The abundance, capacity, and value of cropland jointly determine agricultural output and profit margins in different regions. Agricultural labor, the most active and mobile factor of production, directly affects the allocation of cultivated land resources [44]. Agricultural machinery can effectively replace the labor force, and is also a considerable factor affecting the use of cropland. In this research, the characteristics of agricultural production factors are represented by some specific indexes, including cultivated land per capita, farmland production potential, land price, agricultural employment rates, and agricultural machinery levels.

*Socio-economic development:* Cropland can be reallocated and adjusted due to industrialization and urbanization, and converted to industry and urban sectors with the expansion of cities and towns, resulting in the withdrawal of croplands [35]. The consequent scarcity of croplands also drives up the cost of grain production, which reduces the motivation of farmers to engage in grain production. These rigid constraints on cropland may be detrimental to grain production, causing fluctuations in grain supply and changes in the NGPCL.

*Grain production policy:* Cultivated land is a kind of quasi-public good, which has strong externalities and is subject to policy interventions [15]. By implementing various food production management measures such as increasing subsidies and improving agricultural infrastructure in the major grain producing area, it is able to improve the earnings of grain production, thereby curbing NGPCL. Hence, this study uses the division of the main grain producing areas, main sales areas, and production and sales balance areas to characterize regional policy.

### 3.3. Data Sources

The data applied in this study contain socioeconomic statistics data, land use data, and other online data. The balanced panel dataset include 351 prefecture-level cities in China over the period of 2000–2020 (Hong Kong, Macao, Taiwan, and Tibet are not included because of a lack of data). The data of the cultivated land area, sown area of the grain, population size, agricultural employment, total power of agricultural machinery, and GDP are obtained from the China Statistical Yearbook (2000–2020) and the China Land and Resources Statistical Yearbook (2000–2020), which are available from China’s Economic and Social Bit Data Platform (https://data.cnki.net, accessed on 20 May 2022). Some missing data are interpolated using the average value of the two adjacent years. Multiple cropping index, land use image, DEM data, and farmland productivity potential data come from the Resource and Environment Data Clod Platform of CAS (http://www.resdc.cn, accessed on 20 May 2022). The multiple cropping index uses the maximum multiple cropping potential under the irrigation scenario, which only considers the light-temperature conditions to limit crop growth. The land price is divided by 30 through the “comprehensive land price for expropriated agricultural areas” published by each prefecture-level city considering the annual rent. The policy indicators come from the division of major grain producing areas, production and marketing balance areas, and major grain marketing areas published by the Ministry of Agriculture and Rural Affairs of China (http://www.moa.gov.cn/, accessed on 20 May 2022).

## 4. Results

### 4.1. The Overall Trend of NGPCL in China

**Figure 3:**
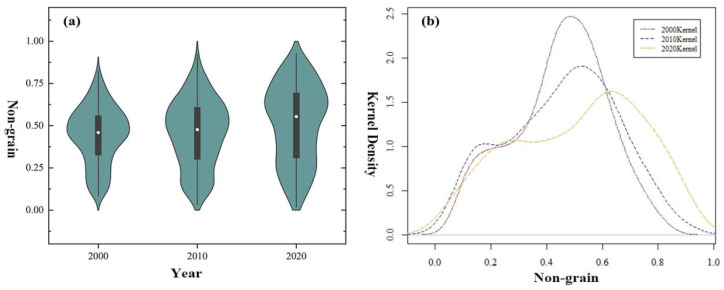
The evolution of NGPCL in China from 2000 to 2020. (**a**) The degree of NGPCL in 2000, 2010, and 2020. (**b**) The kernel density curve of NGPCL in 2000, 2010, and 2020.

The degree of NGPCL in China is on the rise. From 2000 to 2020, the average value of NGPCL at the prefecture-level city in China grew from 0.44 to 0.51 (Figure 3a). The kernel density curve moved to the right gradually, indicating that the level of NGPCL increased in most cities (Figure 3b). The peak value of the curve, which also denotes the modal value of NGPCL, demonstrated a rise from 0.5 in 2000 to about 0.7 in 2020 (Figure 3b). With the Chinese government boosting market economic reform and adjustment of the agricultural structure, grains gave way to economic crops for their relatively lower yielding and returns [51]. As a result, NGPCL has proliferated nationwide in China.

The gap of NGPCL among prefecture-level cities is consistently enlarging. From 2000 to 2020, the minimum value of NGPCL in cities experienced a decrease from 0.08 to 0.01, while the maximum value saw the opposite trend from 0.82 to 0.93, which demonstrated that the range of NGPCL increased from 0.74 to 0.91 (Figure 3a). The wave height of the kernel density curve featured a decrease and the horizontal width widened, indicating that the polarization of NGPCL in China intensified.

### 4.2. The Spatial Pattern of NGPCL in China

**Figure 4:**
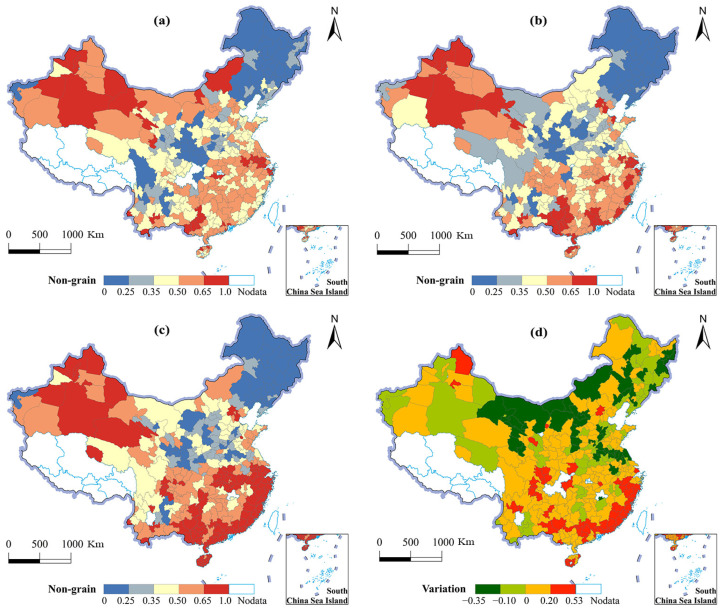
Spatial pattern evolution of NGPCL in China. (**a**) The NGPCL of China in 2000; (**b**) the NGPCL of China in 2010; (**c**) the NGPCL of China in 2020; (**d**) the spatial variation of NGPCL in China from 2000 to 2020.

The level of NGPCL differed obviously in different areas, among which the northwest and southeast were high, while the northeast and central regions were low (see Figure 4). In 2000, there were 91 prefecture-level cities that could be classified as low-value cities (NGPCL < 0.35), which were relatively concentrated in traditional agricultural areas such as the Northeast Plain, Weihe Plain, Jianghan Plain, and Western Sichuan Basin (Figure 4a). There were 139 cities that could be classified as high-value cities (NGPCL > 0.50), which were dispersed in the Gan–Xin desert plateau, southern hilly area, the middle and lower Yangtze River, and in urban agglomerations such as Beijing–Tianjin and the Pearl River Delta. The high NGPCL in the Gan–Xin desert plateau and the southern hilly region could be attributed to the dry climate and unfavorable geographical conditions. Urban agglomeration areas boast a highly-developed economy and diverse market demand, where the profit gap between the grain plantation and non-grain purpose was extremely large. In 2010, the spatial pattern of NGPCL witnessed a slight change (Figure 4b). The low value cities of NGPCL moved northwards and towards the center, while the high value cities continued to be concentrated southward and in the northwest. The Northeast Plain, Western Inner Mongolia Plateau, Loess Plateau, and North China Plain demonstrated a low value of NGPCL, which could be attributed to the further concentration of China’s grain production in the major grain producing areas. The number of high value cities with NGPCL rose to 157, with the Gan–Xin desert plateau remaining high, the southeast coastal and Chengdu-Chongqing urban agglomeration leapfrogging into high value areas, and Beijing–Tianjin, Yangtze River Delta and Pearl River Delta featuring a further increase. In 2020, the NGPCL was also high in the northwest and south, and was low in northeast and central China, with greater spatial heterogeneity (Figure 4c). The low value area of NGPCL extended to Northeast China and the Loess Plateau. The number of high value cities grew dramatically to 195, featuring the pattern of “two high value concentration areas + multiple high value spots”, and were located in the south and northwest China and in several provincial capitals.

In terms of spatial variations, most regions witnessed a growth of NGPCL (Figure 4d). Among them, the southeast coastal economic belt, Guangdong and Guangxi regions, and eastern Sichuan saw the highest increase. Nevertheless, Inner Mongolia, the Northeast Plain, North China Plain, and northwest China exhibited a decreasing trend in NGPCL. On the one hand, climate warming has released the natural constraints in the northern regions, causing the northward moving of grain production in China. On the other hand, the implementation of China’s regional grain production policy acts as a vital factor of NGPCL. The grain production support policy favoring the main grain producing areas, such as northeast China and North China, has fully mobilized farmers’ motivation for grain planting. Conversely, under the open market policy, farmers in developed regions, including the southeast coastal area, widely use cultivated land for non-grain used driven by the high comparative interest.

### 4.3. The Spatial Agglomeration Characteristics of NGPCL in China

**Figure 5:**
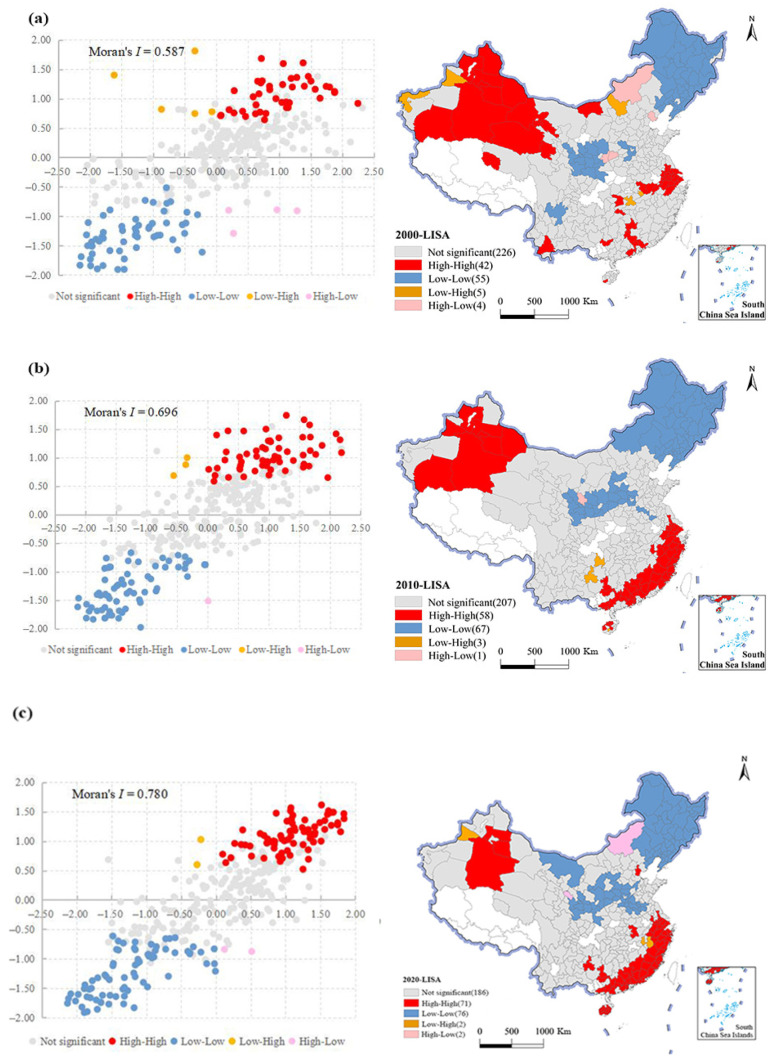
Moran scatter plot of NGPCL in China. (**a**) The Moran scatter plot in 2000; (**b**) the Moran scatter plot in 2010; (**c**) the Moran scatter plot in 2020.

During the period from 2000 to 2020, the Moran’s I index of NGPCL in China increased from 0.587 to 0.780, all of which were significant at a 99% level (Figure 5). The proportion of homogenous agglomerating units (“high–high” and “low–low”) grew from 29.21% to 43.62%, while the ratio of heterogeneous agglomerating units (“high–low” and “low–high”) dropped from 2.71% to 1.19%. These results validate that the spatial agglomeration of NGPCL intensified, the spatial differentiation phenomenon became gradually obvious, and the regional spatial connection was increasingly tight. Influenced by regional subsidy policies and zoning layout plans for advantageous agricultural products, the synergistic advantages of high-grain-producing areas have been further strengthened, and the regional division of grain production has become clearer [15,52]. As a result, the spatial agglomeration of NGPCL has been constantly enhanced.

Most prefecture-level cities with adverse climatic conditions, highly developed economies, and diversified market demands, such as the southeast coastal economic belt, Gansu–Xinjiang Desert plateau, Yangtze River Delta, and Pearl River Delta, presented agglomerations of cities with a low NGPCL (Figure 5). Affected by the capital spillover effect and market radiation effect, local NGPCL exerted a radiative driving impact on adjacent cities, thus forming a “high–high” agglomeration [53]. The “low–low” agglomeration area of NGPCL was mainly situated on the Northeast Plain and Loess Plateau, and expanded to the North China Plain in 2020. Most of the cities in this agglomeration are traditional agricultural areas, which were favored by policy and formed a concentration of grain production, thus becoming a “low–low” agglomeration area.

### 4.4. Influencing Factors of Spatial–Temporal Pattern Change in NGPCL

The aforementioned 11 indicators were selected to analyze the influencing factors of NGPCL in this paper. Firstly, the multicollinearity of the index was tested. The variance inflation factor (VIF) of various driving factors were calculated with STATA 15.0 [54]. The VIF values of all of the factors were all below 10, validating that there were no redundant variables in the model and no multicollinearity among the given factors.

This paper used an ordinary least square model (OLS), spatial lag model (SLM), and spatial error model (SEM) to explore the influencing factors of NGPCL. The R^2^ of all of the regression models was above 0.85, indicating that these models were effective. Two Lagrange multipliers (LM-lag and LM-error) were highly significant, and LMerr (73.61) was greater than LMlag, which suggest that the influencing factors of NGPCL not only included explanatory variables and their terms, but also included some invisible error terms. The above results suggest that the SEM model worked best in this research(see Table 2).

**Figure 6:**
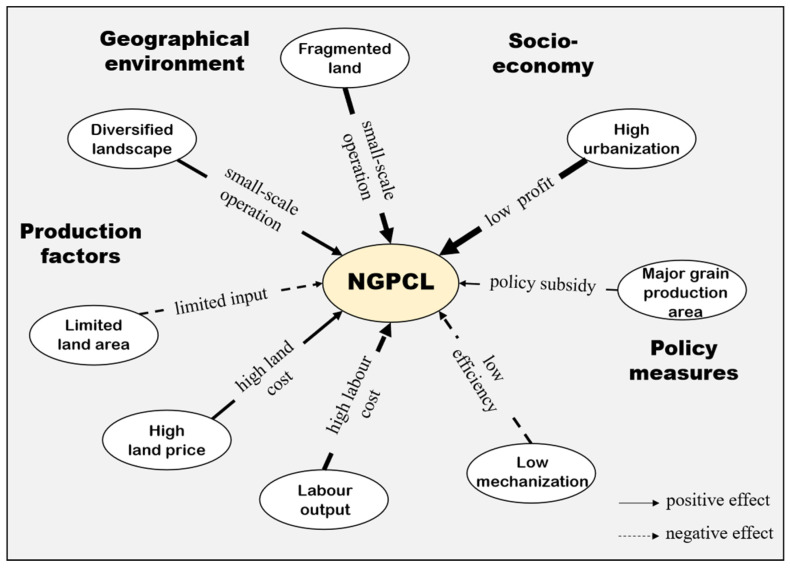
Mechanism of the key factors of NGPCL.

The regression results of the SEM model show that the urbanization rate (0.325), land fragmentation (0.192), landscape diversity (0.070), land price (0.070), and grain production policy (0.025) had a significant positive effect on NGPCL, while the agricultural employment rate (−0.175), agricultural machinery level (−0.041), and cultivated land per capita (−0.026) had significant negative effects on it. Based on the existing analysis framework and the regression results, this paper summarizes the driving mechanism of NGPCL (Figure 6).

Fragmentary and diversified natural background lays the geographical foundation for NGPCL. Fragmented and scattered land hinders grain production on a large scale and provides varied soil and farming conditions for different crops [55]. The diversified landscape assumes the spatial carrier for multifunctional agriculture, so that the cropland is no longer for single grain cultivation, but is instead for multi-functional superposition and cross-mixed use.

Limited agricultural production factors constitute the resource basis of NGPCL. For farmers with tight cultivated land resources, it is hard to raise the planting income through large-scale production [56]. The transfer of labor to non-agricultural industries greatly increases the opportunity cost of agriculture, combined with the high land price, triggering higher production costs. Farmers, therefore, maximize land use profit by using high-quality land for more profitable non-grain purposes. Compared with cash crops, grain crops are more dependent on agricultural machinery. Facing the stretched agricultural labor and rising price of production factors, the restriction of the agricultural machinery input further causes a decrease un grain production efficiency and benefit, which may render a sizeable portion of NGPCL.

The urbanization rate accounts for the largest share explaining the NGPCL. Urban expansion acts as an inducement for the flow and reallocation of agricultural production factors, which raise the cost of grain production. In addition, urbanization also comes with diet structure upgrades that promote diversified land use and agricultural production [57]. As a result, grain returns have declined in comparative terms and plenty of cropland has been converted for non-grain purposes.

Grain production policy serves as a regulatory tool for NGPCL. Supportive grain policies could improve farmer’s income from grain production by various measures, such as increasing grain production subsidies and perfecting agricultural infrastructure. This kind of grain-friendly policy environment plays a vital role in improve farmers’ willingness towards grain production and reduces the tendency of NGPCL.

## 5. Discussion

### 5.1. NGPCL Serves as a Challenge to Food Security in the New Era

NGPCL serves as an important challenge to China’s food security and national development in the new era [10]. The most immediate impact of NGPCL has been to reduce the land area used for grain cultivation, thus putting great pressure on food production. In addition, unreasonable NGPCL in some areas has exacerbated this structural imbalance between the supply and demand for agricultural products, which is not conducive to agricultural development. More seriously, NGPCL causes damage to the plough layer, which leads to an irreversible reduction in the food production capacity. Previous research has shown that some NGPCL types, such as afforestation and digging ponds for farmed fish, will destroy the physicochemical property of soil and exacerbate non-point source pollution, thereby resulting in permanent damage to the tillage layer [58,59,60]. However, the effects of different types of NGPCL vary, and not all are harmful, so their role in food security should be analyzed differently [45]. Some NGPCL types, such as cropland abandonment and fruit and vegetable cultivation, cause little damage to the land, and can meet the needs of rural development and diversified food consumption. Therefore, in the current complex context of climate change, population growth, and rapid urbanization, we need to give more attention to NGPCL. Scientific measures should be taken to control NGPCL in a reasonable range, so as to achieve the comprehensive goal of ensuring food security and economic and social development [21,29].

### 5.2. Control Measures Should Be Matched with the Differentiated Pattern of NGPCL

With economic and social development, the level of NGPCL has increased remarkably; however, this differed obviously among different areas [61]. In this process, the geographical division of labor in food production is further clarified, and the comparative advantages of regional agriculture are highlighted [45]. Therefore, differential control should be applied to the NGPCL in different areas. For high-value agglomeration areas of NGPCL, such as the Gansu–Xinjiang Desert Plateau area and the southeast coastal economic belt, appropriate NGPCL should be allowed. Such areas have a strong market demand and obvious comparative advantages for non-grain products, so they should be encouraged to develop a diversified agriculture, thus serving urban consumption. However, destructive NGPCL should be banned, such as digging ponds for fish and planting trees, so as to avoid damage to the tillage layer. NGPCL should be strictly prohibited in areas with a low concentration of NGPCL, such as the Northeast Plain and the North China Plain. These traditional farming areas, which have a good foundation of cultivation and great advantages for grain production, should be protected in grain planting by increasing subsidies and cultivating diversified agricultural operators, etc.

### 5.3. Targeted Managements Are Required Based on the Perspective of the Human–Land Relationship

Although relevant studies have explored the control measures of NGPCL, they lack reasonable classification and do not clarify the causes of NGPCL, which leads to a weak operability of measures [27]. NGPCL is affected by the background of rapid economic development, and is the result of multiple factors, including a regional geographical environment, input of agricultural production factors, socio-economic development, and grain production policy [62]. Therefore, control measures should be proposed suiting the cause and essence of NGPCL [45]. First of all, in areas with a developed market economy and high level of urbanization, NGPCL should be appropriately relaxed, and diversified urban agriculture should be encouraged, so as to satisfy diversified market demands and promote agricultural economic development. Secondly, it is supposed to vigorously promote land transfer and strengthen machinery input, so as to improve grain production efficiency and profit. Third, many studies advocate that grain subsidies should be increased to make grain growing truly profitable for farmers [61,63]. Fourth, fostering diversified grain production subjects. There are various measures, such as improving the quality of farmers, encouraging the cultivation of family farms, agricultural cooperatives, agricultural companies, and other entities, that can provide labor support for grain production.

In addition, owing to the complexity of NGPCL, the rapid change in socio-economic background, the importance of food security, the research perspective, and the content of NGPCL need to be further expanded. There are some factors impacting food security that need to be given more attention in future research, such as production efficiency [64], modern farming [65], agricultural trade [66], and farmers’ cognition [67], and so on. Thus, NGPCL can be controlled more rationally and comprehensively.

## 6. Conclusions

This study constructed a framework to measure NGPCL, revealed the spatial–temporal pattern of NGPCL at the prefecture level, and then analyzed its driving factors from the perspective of the human–land relationship. Overall, there are four main conclusions from our study.

Firstly, the overall degree of NGPCL in China showed an upward trend, with the average value of municipalities increasing from 0.44 to 0.51. However, the range in value of NGPCL among municipalities rose from 0.74 to 0.91, indicating that the gap in NGPCL in China has been remarkably enlarged.

Secondly, the spatial pattern of NGPCL is high in the northwest and southeast, and low in the northeast and central regions. The southern economic developed area exhibited the highest increase, while the Inner Mongolia, northwest China, and traditional agricultural areas experienced a decreasing trend in NGPCL.

Thirdly, the spatial agglomeration of NGPCL has been intensified, which demonstrates that the geographical division of grain production was strengthened. The Gansu–Xinjiang Desert plateau, southeast coastal economic belt, and urban agglomeration areas exhibited “high–high” agglomeration, while the traditional agricultural areas exhibited “low–low” agglomeration.

Fourthly, NGPCL is positively correlated with the urbanization rate, land fragmentation, landscape diversity, land price, and grain production policy, while it is negatively linked with the agricultural employment rate, agricultural machinery level, and cultivated land per capita. Fragmentary and diversified natural background lays the geographical foundation for NGPCL, and limited agricultural production factors constitute the resource basis. Urbanization rate dominates the process of NGPCL, while grain production policy functions as a vital external regulatory tool.

It is of great importance to rationally control the NGPCL. On the one hand, targeted management is required based on the perspective of the human–land relationship, such as strengthening the machinery input, perfecting the grain subsidy system, and so on. On the other hand, control measures should be matched with the differentiated pattern of the NGPCL, so as to balance the market demand satisfaction and cultivated land protection.

On the basis of this study, detailed future research could be conducted at a fine scale and be dedicated to distinguishing NGPCL types. At the same time, there are some factors at the plot scale, and the farmer’s point of view should be considered in order to understand the essence of NGPCL. Last, but not least, more research is needed to quantitatively and multidimensional assess the impact of NGPCL on food security and ecosystems.

## Figures and Tables

**Table 1:** Description of the influencing factors.

Variables	Variable Description	References	Mean	Std	Min	Max
Slope (*AS*)	Percentage of area with a slope greater than 15°	[21,45]	0.12	0.22	0.02	0.93
Cultivated land fragmentation (*CF*)	Mean value of homogenization of the mean patch area and patch density	[46,47]	0.43	0.15	0	0.74
Landscape diversity (*LD*)	Shannon’s Diversity Index	[47]	1.67	0.46	0.64	2.44
Cultivated land per capita (*CP*)	Per capita cultivated area calculated by rural population (hm^2^/per)	[14,21,48,49]	0.36	0.66	0.01	5.08
Farmland production potential (*FPP*)	kg/ha	[30,47]	2732.72	2221.40	0.07	8367.65
Land Price (*LP*)	The average of the comprehensive land price of the regional levy area divided by 30 (yuan)	[30]	1966.24	1072.93	332.02	8683.33
Agricultural employment rates (*AER*)	Proportion of agricultural workers in rural (%)	[36,50]	0.45	0.16	0.05	0.87
Agricultural machinery level (*AM*)	Agricultural machinery power per unit cultivated area (kW·h/hm^2^)	[9,30,50]	948.73	539.47	64.50	2727.39
GDP Per Capita (*PGDP*)	yuan per person	[14,21,45]	50,516.21	25,530.03	14,256	189,309
Urbanization rate (*UR*)	%	[14,21,36]	0.55	0.16	0.28	0.98
Policy (*PO*)	Main grain producing area = 1; Production and marketing balance area = 2; Main grain marketing area = 3	[21]	1.64	0.77	1	3

**Table 2:** Results of the spatial econometric model.

Variables	OLS	SLM	SEM
*AS*	0.015 (0.392)	0.009 (0.262)	−0.071 (−1.500)
*CF*	0.172 (3.026) ***	0.139 (2.626) ***	0.192 (3.673) ***
*LD*	0.100 (6.210) ***	0.088 (5.842) ***	0.070 (4.791) ***
*CP*	−0.015 (−1.802) *	−0.019 (−2.131) *	−0.026 (2.389) *
*FPP*	−0.011 (−0.597)	−0.009 (−0.510)	−0.015 (−0.522)
*LP*	0.078(3.402) *	0.069 (3.237) ***	0.061 (2.835) ***
*AER*	−0.245 (−5.825) ***	−0.191 (−4.818) ***	−0.175 (−4.887) ***
*AM*	−0.048 (−4.130) ***	−0.054 (−4.950) ***	−0.041 (−3.905) ***
*PGDP*	−0.009 (−0.595)	−0.008 (−0.564)	0.018 (1.243)
*UR*	0.332 (4.748) ***	0.297 (4.568) ***	0.325 (5.427) ***
*PO*	0.009 (1.057)	−0.001 (−0.186)	0.025 (2.040) *
W-Y		0.188 (6.073) ***	
Lambda			0.601 (12.085) ***
R^2^	0.852	0.868	0.889
Log-L	355.153	373.374	387.318
AIC	−686.307	−720.748	−750.637
SC	−640.289	−670.896	−704.620

Note: Standard deviations are shown in brackets. *, *** denote 10% level of significance and 1% level of significance, respectively.

## Data Availability

Data are available in a publicly accessible repository.
